# Adherence to the Mediterranean diet can beneficially affect the gut microbiota composition: a systematic review

**DOI:** 10.1186/s12920-024-01861-3

**Published:** 2024-04-17

**Authors:** Armin Khavandegar, Ali Heidarzadeh, Pooneh Angoorani, Shirin Hasani-Ranjbar, Hanieh-Sadat Ejtahed, Bagher Larijani, Mostafa Qorbani

**Affiliations:** 1 Non-Communicable Disease Research Center, Alborz University of Medical Sciences, Karaj, Iran; 2 Sina Trauma and Surgery Research Center, Tehran University of Medical Sciences, Tehran, Iran; 3 Endocrinology and Metabolism Research Center, Endocrinology and Metabolism Clinical Sciences Institute, Tehran University of Medical Sciences, Tehran, Iran; 4 Obesity and Eating Habits Research Center, Endocrinology and Metabolism Clinical Sciences Institute, Tehran University of Medical Sciences, Tehran, Iran

**Keywords:** Mediterranean diet, Dietary intervention, Gut microbiota

## Abstract

**Aim:**

Dietary patterns could have a notable role in shaping gut microbiota composition. Evidence confirms the positive impact of the Mediterranean diet (MD), as one of the most studied healthy dietary patterns, on the gut microbiota profile. We conducted this systematic review to investigate the results of observational studies and clinical trials regarding the possible changes in the gut microbiota composition, metabolites, and clinical outcomes following adherence to MD in healthy cases or patients suffering from metabolic disorders.

**Methods:**

A systematic literature search was conducted on PubMed, Web of Science, and Scopus databases until October 2023. Two researchers separately screened the titles, abstracts, and then full-text of the articles and selected the relevant studies. Quality assessment of observational and interventional studies was performed by Newcastle-Ottawa and Cochrane checklists, respectively.

**Results:**

A total of 1637 articles were obtained during the initial search. Ultimately, 37 articles, including 17 observational and 20 interventional studies, were included in this systematic review. Ten observational and 14 interventional studies reported a correlation between MD adherence and microbiota diversity. *Faecalibacterium* and *Prevotella* were the most frequent bacterial genera with increased abundance in both observational and interventional studies; an Increment of *Bacteroides* genus was also reported in observational studies. Better glycemic control, lowering fat mass, better bowel movement, decreased bloating, inflammation, and hospitalization risk were the reported clinical outcomes.

**Conclusion:**

Adherence to the MD is associated with significant beneficial changes in the gut microbiota diversity, composition, and functions and major clinical improvements in most populations.

## Introduction

Gut microbiota is a complex dynamic microbial system helping in better gastrointestinal function [1]. Although the microbiota concept is not yet fully understood, it is known that millions of bacteria colonized the human intestines, contributing to its formation. The gut microbiota composition extensively affects human health, and each individual's dietary intake plays a major role in the microbiota composition. The interactions between diet and gut microbiota are observed to be mutual [2]. Growing evidence shows that the gut microbiota composition extensively affects the host's immunological, nutritional, and metabolic functions and plays a critical, symbiotic role in human health [3–5].

It is observed that the dietary pattern could have a notable role in shaping the gut microbiota composition by providing substrates that can differentially promote the growth of specific microbes and communities [6]. Mediterranean diet (MD) is one of the most studied healthy dietary patterns, characterized by high amounts of fruits, vegetables, nuts, seeds, olive oil, and unrefined grains, moderate quantities of fish, a small amount of poultry, and least possible consumption of red and processed meats [7].

Evidence from the literature illustrates a beneficial effect of MD on metabolic and chronic diseases, including obesity, type-2 diabetic mellitus, cardiovascular disease, and metabolic syndrome, which may be partly through beneficial changes in gut microbiota composition and function [8–10]. A high proportion of plant-based foods in MD correlates with a higher percentage of short-chain fatty acids (SCFAs) and fiber-degrading bacteria in the feces [11]. It has been shown that subjects with higher adherence to MD had a lower presence of *E. coli* and an increased total abundance of bacteria, a higher *Bifidobacteria* to *E. coli* ratio, and an increased prevalence of *C. Albicans* [1].

Bacteria ferment dietary fiber in the colon to produce SCFA, which is believed to have systemic anti-inflammatory effects [12]. Moreover, polyphenols in MD are known to have prebiotic actions that can change gut microbiota and produce metabolites with consequential effects on host health [13]. The effects of MD on the gut microbiota composition have been widely investigated in different studies, but evidence from individual studies is somehow inconsistent. In some studies, higher adherence to MD has resulted in positive gut microbiota diversity [14, 15], yet some evidence reported no change or even decrease of some beneficial bacterial phyla after MD intervention [16, 17].

A wide range of studies targeting different populations have been conducted in this regard; thus, defining the appropriate criteria and summarizing the findings can be challenging. Hence, we conducted this systematic review to investigate the results of observational studies and clinical trials regarding the possible changes in the gut microbiota diversity and abundance, its metabolites, and finally, participants' clinical outcomes following adherence to MD in healthy populations or patients suffering from metabolic disorders.

## Methods and materials

### Search strategy and selection of studies

A systematic literature search was conducted on PubMed, Web of Science, and Scopus databases. All related articles published up to October 2023 were considered for inclusion. Besides, Google Scholar and recent review articles' references were checked for further article inclusion. Search queries were as following: ("Mediterranean diet"[Title/Abstract] OR "Mediterranean dietary pattern"[Title/Abstract] OR "Mediterranean dietary intervention"[Title/Abstract] OR "Mediterranean-style diet"[Title/Abstract]) AND ("microbiota"[Title/Abstract] OR "microbiome"[Title/Abstract] OR "microflora"[Title/Abstract] OR "microbial profile"[Title/Abstract] OR "microbial composition"[Title/Abstract] OR "bacterial load"[Title/Abstract]).

The method of presenting the topics, including analysis and interpretation, determining the study's objectives, and collecting the findings, was performed based on the preferred reporting items for systematic reviews and meta-analyses (PRISMA) [18].

### Eligibility criteria

Two researchers separately screened the titles, abstracts, and then full-text of the articles and selected the relevant studies based on their relevance to the objectives of the systematic review, separately. Disagreements between the two researchers were resolved by discussion or consulting with a third reviewer until reaching a consensus. Duplicate papers retrieved from different queries were removed, and only articles with more complete data were considered. Studies were excluded if the main text was not available or was not in English, if the articles did not investigate the gut microbiota composition following adherence to MD, or if a dietary intervention was not described as MD by the article's authors.

Articles conducted on healthy subjects or patients with metabolic disorders were included in our study. Patients with an inflammatory disease, including Inflammatory Bowel Disease (IBD) and Rheumatoid Arthritis (RA), were excluded from our study. Gut microbiota alterations with/without clinical change following MD were considered outcomes. Reviews, protocols, editorials, letters, case reports, and experimental or animal studies were excluded. Therefore, only observational and interventional studies with original data on humans were included in the present study.

### Data extraction

The extraction checklist for both observational and interventional studies consisted of the following parts: Surname of the first author, publication year, country, information on the study design, participants' characteristics (age, gender, and ethnicity), study duration, study cohort, sample size, alpha and beta diversity, microbial alteration, gut microbiota-derived metabolites, and clinical outcomes. For interventional studies, dietary intervention, randomization procedure, blinding of measurements, compliance with the interventions, and baseline and post-intervention gut microbiota composition were added to the checklist. For observational studies, the dietary assessment method was added to the checklist.

The full text of the papers was checked to retrieve the relevant information. The primary outcome to be investigated in this review was the effect of the Mediterranean diet on gut microbiota composition (bacterial abundance and diversity) and microbiota-derived metabolites. Secondary outcomes were the effects of MD on clinical outcomes, including prevention and treatment of weight gain and obesity, hyperglycemia, insulin resistance, inflammation, and dyslipidemia.

### Quality assessment of studies

Risk of bias assessment was accomplished by one author (A. Kh.); afterward, an accuracy check was performed by another author (H-S. E.). Interventional studies' quality was assessed using the Cochrane risk of bias tool [19]. Cochrane risk of bias tool consisted of six domains: random sequence generation, allocation concealment, blinding of participants and personnel, blinding of outcome assessment, incomplete outcome data, selective reporting, and other biases. Each interventional study was categorized as high, medium, and low risk.

The quality of observational studies was assessed using an adapted New-Castle Ottawa Scale (NOS) tool for cross-sectional and cohort studies which was developed to assess the quality of non-randomized studies [20, 21]. The adopted versions of NOS consist of three bias-evaluating sections: Selection, Comparability, and Outcome. Each section consists of further subsections, differing in two adopted NOS versions. High-quality articles were defined as $\ge 7$ stars, medium (4-6 stars), and low (0-3 stars).

## Results

### Overview

**Fig. 1:**
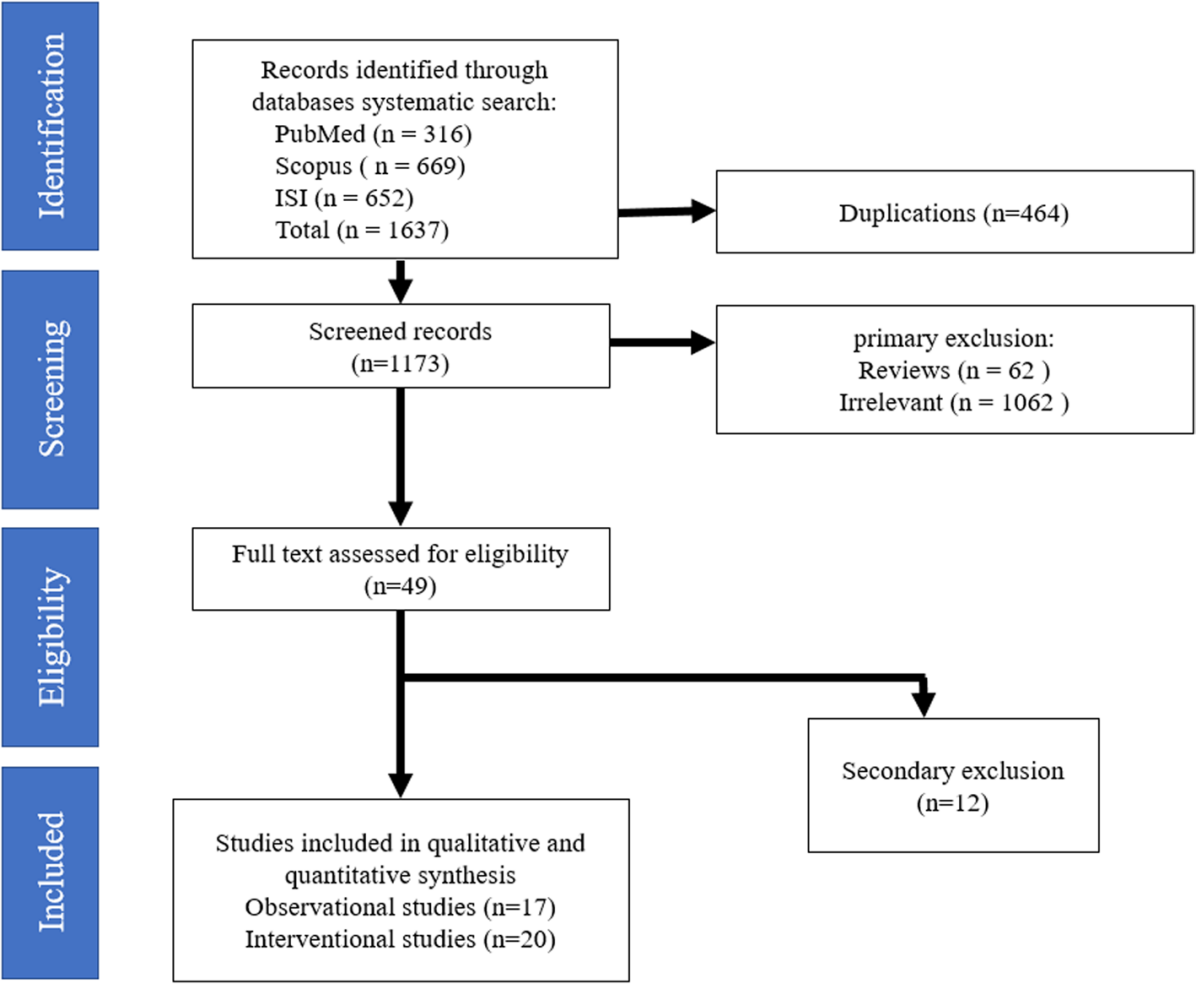
The Preferred Reporting Items for Systematic Reviews and Meta-analysis (PRISMA) for included articles in the current study

A total of 1637 articles were obtained during the initial search (PubMed, Scopus, Web of Science, hand searching), of which 464 were deleted due to duplication, 1136 records did not meet the inclusion criteria or were inappropriate due to indirect relevance, or missing outcome data were also removed. Ultimately, 37 articles (17 observational and 20 interventional studies) successfully met the search criteria and were included in this systematic review (Fig. 1). The findings of these articles are summarized in Tables 1 and 2.

**Table 1:** Characteristics of the included observational studies

**number**	**First Author, Year of publication**	**Study design**	**Sample size(n)**	**Region**	**Participants characteristics**	**Microbiota assessment**	**Outcome (Significant Difference in Microbiota Composition)**	**Clinical outcomes**	**Bacterial Diversity and correlation with MD adherence**	**Main** **Nutrients features**
						**Metabolites assessment**	**Outcome (Difference in metabolites)**			
						**Questionnaire for dietary adherence assessment**				
**1**	**Gutierrez-Diaz (2016) **[17]	cohort	31	Italy	Healthy individual without any PMH, and no DH in the past 6 months. mean age: 42.1 y; 8M, 24F BMI: 26	16S rRNA sequencing	*↑* Bacteroidetes* (p=0.001)* *↑ Prevotella (p=0.003)* *↑ Prevotellacea (p=0.002)* ***↓*** Firmicutes* (p=0.003)* ***↓**** Lachnospiraceae (p=0.045)*	NA	NA	*↑* Cereals *↑* Legumes *↑* Vegetable *↑*fruits *↑*monosaturated to saturated ratio $\to$ ethanol ***↓*** meat ***↓*** milk
						MS; HLPC; GC; CE	faecal propionate (*p*=0.034) fecal butyrate (*p*=0.018)			
						• (0-8) points Trichopoulou MDS • FFQ				
**2**	**Gutierrez-Diaz (2017) **[22]	Cross-sectional	74	Spain	Healthy individual >50 years without any PMH, and no DH (including probiotics) in the past month, mean age: 71.3 y 20M, 54F	16S rRNA sequencing	*↑ Clostridium cluster XIVa (p=0.016)* $\uparrow$ *Faecalibacterium* (*p*=0.006)	NA	NA	*↑* Cereals *↑* Legumes *↑* Vegetable *↑*fruits *↑*monosaturated to saturated ratio $\to$ ethanol ***↓*** meat ***↓*** milk
						MS; HLPC; GC; CE	↑ benzoic acid (*p*<0.05) ↑ 3-hydroxyphenylacetic acids (*p*<0.05); No effect (*p*>0.05) on phenylacetic acid, phenylpropionic acid, 3-(3-hydroxyphenyl) propionic acid, 4 hydroxyphenyl acetic acid, vanillic acid, syringic acid, phthalic acid or γ-valerolactone.			
						• (0-8) points Trichopoulou MDS • FFQ				
**3**	**Mitsou 2017 **[1]	Cross-sectional	116	Greece	Healthy individual 18-65 years without any PMH, and no DH (including probiotics) Mean age: 42 y 61M, 55F BMI: 27	16S rRNA sequencing	*↑ Bacteroides (p=0.011)* *↓ Escherichia coli (p=0.022)* *↓ Candida Albicans (p=0.039)*	↑fecal moisture& defecation frequency ↓ bloating	NA	*↑* non-refined *↑* cereals *↑* fruit *↑*vegetables *↑* potatoes *↑* legumes *↑* olive oil *↑* fish
						GC	↑ acetate (*p*=0.009) ↓ Caproic acid (*p*=0.045); No effect (*p*>0.05) on total SCFA, propionate, butyrate, iso-butyrate, iso-valerate, iso-caproic acid, valerate and heptanoic acid			
						• Three tertiles (0-11 points) • Panagiotakos classification MDS • FFQ				
**4**	**Shankar2017 **[23]	cohort	42	USA & Egypt	Healthy teenagers without any PMH, and no DH in the last 3 months (including probiotics); 28 Egypt (mean age: 13.9 y) received MD 14 U.S (mean age= 12.9 y) received western Diet	16S rRNA sequencing	*↑ Prevotella (p<0.05)* *↓ Bacterioides* (*p*<0.05)	NA	Bray-Curtis **beta** diversity: significant intersample dissimilarity	*↑* fruits *↑*vegetables *↑* whole grain *↑* beans *↑* nuts *↑* plant fats ↓ meats ↓ sweets
						nuclear magnetic resonance	↑ SCFA (*p*<0.05) (acetatae; butyrate; propionate)			
						• Not specified MDS				
**5**	**Bowyer 2018 **[24]	cohort	2070	UK	mono- and dizygotic twins; 1863 F, 207 M mean age: 60.5 mean BMI: 25.9	16S rRNA sequencing	*↓ Ruminococcus (p<0.05)* *↓ Lachnospira (p<0.05)* *↓ Actinomyces (p<0.05)*	NA	**Beta**-diversity: Weighted and Unweighted UniFrac distances/ significant distinction **Alpha** diversity via Chao1, OTUs, Shannon, and Simpson./ Significant correlation of all alpha measures with at least one dietary measure.	NA
						NA	NA			
						• (0-10) points classification MDS, • FFQ • Healthy eating index (HEI), • Healthy food diversity (HFD)				
**6**	**Garcia-Mantrana 2018 **[25]	cross- sectional	27	Spain	Healthy individuals without any PMH, and no DH in the last 2 months (including probiotics); mean age: 39.5 y 16 F (mean BMI:21.95), 11M (mean BMI: 25.29)	16S rRNA sequencing	↑*Christensenellaceae* (*p*<0.05) ↓*Streptococcaceae* (*p*>0.05) ↑ *Bifidobacteriae* (*p*<0.05)	NA	**Alpha** diversity via Chao1 and Shannon./ no significant result in Shannon; but higher bacterial richness via Chao1in MD	NA
						HPLC	↑ total SCFA (*p*=0.020) ↑ acetate (*p*=0.006) ↑ propionate (*p*=0.016)			
						(0-14) points classification MDS, FFQ PREDIMED test				
**7**	**Maskarinec 2019 **[15]	Cohort	1735	USA	Japanese American, Latino, Hawaiians, and African Americans; Healthy; 858 M, 877F, mean age= 69 y 29.3% NL weight, 40.4% overweight, 30.3% obese BMI= 28 [17.1-49.8]	16S rRNA sequencing	*↓* Actinobacteria* (p<0.05)*	NA	**Beta**-diversity: Weighted and Unweighted UniFrac distances/ significant distinction **Alpha** diversity via Shannon./ Linear trend in 4 dietary measure.	NA
						NA	NA			
						(0-8) points Trichopoulou MDS qFFQ HEI				
**8**	**Cox 2020 **[14]	cohort	296	USA and Turkey	200 M; 96 F; Age: 58 BMI: 27 139 Turkish (46 healthy controls, 50 compensated & 43 decompensated cirrhotic; 79M, 60F) received MD 157 American (48 healthy controls, 59 compensated & 50 decompensated cirrhotic; 121M,36F) received western diet	16S rRNA sequencing	↑beneficial taxa (*Oscillibacter, Blautia*) (*p*<0.05),	Altered diet‐microbial interactions could affect the 90‐day hospitalization risk	**Alpha** diversity via Shannon./ higher alpha diversity with MD adherence.	NA
						nuclear magnetic resonance (NMR) spectroscopy	↑ plasma lactate (*p*<0.001)			
						Not specified MDS; FFQ				
**9**	**Gallè 2020 **[26]	Cross-sectional	140	Italy	Apparently healthy 68M, 72F mean age :22.5 y mean BMI:22.4	16S rDNA sequencing	↑ Firmicutes (p = 0.001) ↑ Bacteroidetes (*p* = 0.001) *↑ Lactobacillus(p=0.002)* *↑ Lactococcus (p=0.01)* *↓ Paraprevotella* (*p* = 0.001) *↓Oscillospira* (p = 0.001) *↓Ruminococcus* (*p* = 0.001)	NA	**Beta**-diversity: Bray-Curtis/ significant distinction **Alpha** diversity via Shannon./no significant association	NA
						NA	NA			
						• (0-9) points Martínez-González MDS • IPAQ				
**10**	**Maldonado-Contreras 2020 **[32]	Cross-sectional	20	USA (Caribbean Latino)	Caribbean Latino from senior center with CVD with no antibiotic in last 6 months; 9 with T2DM mean age: 62.7 y mean BMI: 28.9 4M, 16F	16S rRNA sequencing	*↑ Prevotella copri (p=0.001)*	NA	**Beta**-diversity: Uni-Frac / significant association with some of dietary components. **Alpha** diversity via Faith’s PD and Shannon./ significant correlation between Shannon and total HEI-2015 score; no significant association between Faith’s PD and total HEI-2015 score	*↑*fruit *↑*vegetables ↓seafood protein ↓total protein
						MS	↓Acetate in MDS (*p*=0.04) ↓ butyrate in MDS (0.03) ↑Acetate in HEI (*p*=0.06) ↑ propionate in HEI (0.02)			
						• (0-9) points Trichopoulou MDS • HEI • FFQ • DAS-28				
**11**	**Ruiz-Saavedra 2020 **[27]	cross-sectional	73	Spain	Healthy individuals without any PMH, and no DH in the last 2 months (including probiotics) 20 M, 53 F BMI:19.9-37.5 Age:56-95	PCR	*↑Faecalibacterium. Prausnitzii (p<0.05)* *↓ Lactobacillus. Spp (p<0.05)*	*↓* serum IL-8	NA	NA
						Gas chromatography	↑ SCFA (*p*-value<0.05)			
						• (0-9) points Trichopoulou MDS • FFQ • DII • EDII • HEI • AHEI • DQI-I • MMDS • rMDS				
**12**	**Valeriani 2020 **[28]	Cross- sectional	59	Italy (*Caucasian)*	Healthy individuals without any PMH, and no DH in the last 3 months; mean BMI:22 mean age:23 y 29M, 30F	16S rRNA sequencing	↑Firmicutes (*p*>0.05) ↓Bacteroidetes (*p*>0.05)	NA	NA	NA
						NA	NA			
						• (0-9) points Martínez-González MDS • IPAQ				
**13**	**Rosés 2021 **[29]	Cohort	360	Spain	Healthy individual without PMH and DH with BMI:25-40; 251F, 109 M mean age: 45.0 y mean BMI: 28.8	16S rRNA sequencing	*↑Oscillibacter valericigenes (p<0.001)* *↑Oscillospira (Flavonifractor) plautii (p<0.001)* *↑Roseburia faecis (p<0.001)* *↑Ruminococcus bromii (p=0.01)* *↑Butyricicoccus pullicaecorum (p<0.001)* *↑Papillibacter cinnamivorans (p=0.04)* *↑ Bifidobacterium animalis (p<0.001)*	NA	**Beta**-diversity: Bray-Curtis/ no significant correlation with MD adherence. **Alpha** diversity via Shannon./no significant correlation with MD adherence.	*↑* Fiber *↑* Legumes *↑*Vegetables *↑* fruit *↑* olive oil *↑* nuts
						Reconstructive method by Kegg	↑SCFA (*p*-value: N/A) (SCFAs were not directly quantified via fecal/blood samples. They were assessed indirectly through other biomarkers, such as K0)			
						• (0-14) points classification MDS; • FFQ including 137 food items with corresponding portion size, • PREDIMED 14-item questionnaire				
**14**	**Wang 2021 **[30]	prospective	307	USA	Healthy at baseline Age:45-80 y 307M	16sr RNA sequencing	*↑ Eubacterium eligens (p<0.05)* *↑ Faecalibacterium prausnitzii (p<0.05)* *↑ Bacteroides cellulosilyticus (p<0.05)* *↓ Clostridium leptum (p<0.05)* *↓Collinsella aerofaciens (p<0.05)* *↓ Ruminococcus torques (p<0.05)*	NA	**Beta**-diversity: Bray-Curtis/ no significant correlation with MD adherence. **Alpha** diversity via Shannon./no significant correlation with MD adherence	*↑*vegetables*↑*legumes, *↑*fruit, *↑*nuts, *↑*whole grains, *↓*red/processed meat, *↑*fish, *↓*alcohol *↑* ratio of monounsaturated to saturated fat.
						*NA*	*↑SCFA* *(p-value: N/A)*			
						• (0-9) points Trichopoulou MDS; • FFQ • MD index; Med-diet				
**15**	**Turpin 2022 **[31]	cohort	2289	Canada	Healthy first-degree relatives of patients with Crohn’s disease; Median age: 18 1083 M, 1206 F	Stool analysis (not specified)	↑*Ruminococcus* (*p*<0.05) ↑*Faecalibacterium* (*p*<0.05)	NA	NA	*↑*fruits, *↑*vegetables, *↑*plant proteins, *↑*whole grains *↑*low-calorie starches *↑*low-fat or no-fat dairy content *↑*milk alternatives
						NA	NA			
						• (0-14) points MDS • FFQ				
**16**	**De Filippis 2015 **[11]	cohort	153	Italy	Apparently healthy volunteers comprising 51 vegetarians, 51 vegans and 51 omnivores; Age: 27-47; BMI: 22	16S rRNA sequencing	*↑ Prevotella (p<0.01)* *↑ Lachnospira (p<0.01)* *↓ L-Ruminococcus* (*p*<0.001)	NA	**Beta**-diversity: Uni-Frac / no significant correlation with MD adherence. **Alpha** diversity via not-determined method./no significant correlation	↑cereals, ↑fruit, ↑vegetable ↑legumes
						GC-MS	↑ total SCFA (*p*<0.05) ↑Acetate (*p*<0.05) ↑ Propionate (*p*<0.05)			
						• (0-9) points Trichopoulou MDS;				
**17**	**Calabrese 2023 **[33]	Cohort	46	Italy	46 moderate to severe NAFLD patients; BMI $\ge$ 25; Age>30 and <60; two groups: Only physical activity and physical activity+MedDiet;	16S rRNA sequencing	*↑Peptococcaceae(p<0.05)* *↑Rikenellaceae (p<0.05)* *↑Oscillospiraceae(p<0.05)* *↑Ruminococcaceae (p<0.05)* *↑Lachnospiraceae (p<0.05)* *↑Haemophilus (p<0.05)* *↑Sanguibacteroides (p<0.05)* *↑Catenibactrium (p<0.05)*	Better DM/hyperlipidemic state control	NA	saturated fats $\le$ 10% of total daily calories
						GC-MS	*↓*Butanoic Acid (*p*<0.05) *↓*Pentanoic Acid (*p*<0.05) *↓*Heptanoic Acid (*p*<0.05)			
						• FFQ				

*MD *Mediterranean Diet, *FM *Fibromyalgia, *RA *Rheumatoid Arthritis, *PMH *Past Medical History, *MS *Mass Specterometry, *MDS *Mediterranean Diet Score, *SCFAs *Short Chain Fatty Acids, *M *Male, *F *Female, *DH *Drug history, *GI *Gastro-Intestinal, *FFQ *Food Frequency Questionnaire, *HPLC *High performance liquid chromatography, *CRP *C-Reactive Protein, *DAS-28 *Disease activity score on 28 joints, *IPAQ *International Physical Activity Questionnaire, *HEI *Healthy Eating Index, *CVD *Cardiovascular Disease, *T2DM *Type-2 Diabetic Mellitus, *DII *Dietary inflammatory index, *EDII *Empirical Dietary Inflammatory Index, *AHEI *Alternative Healthy Eating Index, *DQI-I *Mediterranean adapted Diet Quality Index-International, *MMDS *Modified MD Score, *rMDS *relative MDScore, *IPAQ *International Physical Activity Questionnaire, *NAFLD *Non Alcoholic Fatty Liver Disease, *GC *Gas Chromatography

**Table 2:** Characteristics of included interventional studies

**No**	**First Author, Year**	**Study design**	**Sample size(n)**	**Region**	**Participants** **characteristics**	**Dietary intervention**	**Microbiota assessment**	**Significant Difference in Microbiota Composition**	**Clinical outcomes**	**Microbial diversity and its correlation with MD adherence**	**Main** **Nutrients** **features**
							**Metabolites assessment**	**Significant Difference in metabolites**			
							**Questionnaire for dietary adherence assessment**				
**1**	**Kong et al. 2014 **[43]	Clinical trial	59	France	45 overweight and obese subjects (6 M, 39 F); (mean BMI: 33.2±0.55) 14 healthy females as reference group Mean BMI: 22.62	Three clusters with 7-day dietary records; Cluster 1 with the least healthy eating behavior (*n*=14), Cluster 3 the healthiest eating behavior (*n*=13), and Cluster 2 was in-between clusters 1 and 3 in terms of healthfulness (*n*=18)	qPCR, Metagenomic sequencing	no significant difference across the clusters (*p*>0.05)	↓ hsCRP and IP10 ↑ HAM56+cells	the healthiest dietary cluster had the highest microbial gene richness	↓ confectionary and sugary drinks, ↑ Fruits, yogurts and soups
							NA	NA			
							NA				
**2**	**Haro et al. 2015 **[44]	Prospective randomized controlled trial	20	Spain	20 male patients with obesity Mean age: 63.3 y Mean BMI: 32.2	Two randomized groups first receiving Med diet (35% fat, 22% monounsaturated) and second receiving LFHCC diet (28% fat, 12% monounsaturated) for one year	16S rRNA sequencing	*↓Prevotella (p=0.028)* *↑Roseburia (p=0.002)* *↑Oscillospira (p=0.016)* *↑Parabacteroides* ↑*P. distasonis* (*p*=0.025)	Protective effects on the development of type two diabetes by increasing in the insulin sensitivity measured by OGTT	**Alpha** diversity via Chao1/ no significant correlation **Beta** diversity via UniFrac/ no significant correlation	NA
							NA	Changes in 7 out of 572 metabolites in feces and 3 out of 697 metabolites in plasma were related to the changes in bacterial species			
							NA				
**3**	**Haro et al. 2016 **[37]	Prospective controlled trial	239	Spain	239 patients with CHD with last coronary event over last six months in two groups; 138 metabolic syndrome patients, 101 healthy individuals	two healthy diets: a MD and a LFHCC, for two years in the gut microbiota of MetS patients and those in the control group	16S rRNA sequencing	*↑Parabacteroides* *↑Bacteroides* *↑Faecalibacterium* *↑Bifidobacterium* ↑*P. distasonis* *↑B. thetaiotaomicron* *↑F. prausnitzii* *↑B. adolescentis* *↑B. longum* (*p*-values<0.05)	NA	NA	NA
							NA	NA			
							14-item food questionnaire				
**4**	**Haro et al. 2017 **[38]	Prospective randomized controlled trial	106	Spain	106 subjects with CHD with last coronary event over last six months in three groups; 33 obese men with severe metabolic disease; 32 obese men without metabolic diseases; 41 non-obese men	differences in bacterial community at baseline and after 2 years of dietary intervention following consumption of two healthy diets; MD and low-fat	16S rRNA sequencing	*Bacteroides↑* *Prevotella↑* *Faecalibacterium↑* *Roseburia↑* *Ruminococcus↑* (*p*-values<0.05) *P. distasonis↑ (p=0.014)* *F. prausnitzii↑* (*p*=0.043)	NA	**Alpha** diversity via Chao1 and Faith’s PD/ no significant correlation **Beta** diversity via UniFrac/ no significant correlation	*↑*vegetables, *↑* fruit, *↑* cereals, *↑*potatoes, *↑*legumes, *↑* dairy products,
							NA	NA			
							14-item questionnaire				
**5**	**Djuric et al. 2017 **[41]	Randomized dietary intervention trial	93	USA	Healthy individuals at increased risk of colon cancer as defined;	Participants were randomized to MD / Healthy Eating diet; Biopsy data was available from 88 participants at baseline and 82 participants after six months	16S rRNA sequencing	No significant changes in colonic mucosal bacterial community (*p*>0.05)	NA	**Alpha** diversity via Shannon and inverse Simpson/ no significant correlation **Beta** diversity via community distance index / no significant distinction between control and case group. **Only significant within healthy arm after 6 months dietary intervention**	30% of calories from fat as polyunsaturated: saturated: monounsaturated fatty acids (PUFA: SFA: MUFA) ratio of 1:2:5.
							NA	NA			
							NA				
**6**	**Luisi et al. 2019 **[45]	Randomized controlled trial	18	Italy	Healthy controls (6M, 12 F) Mean BMI: 21.6, Mean age: 41.4 Overweight individuals (11M, 7 F) Mean BMI: 30.152 Mean age 52.1 y	18 overweight/obese subjects (BMI ≥25) and 18 normal weight controls (BMI 18.5–24.9) were fed with MD enriched for three months. Feces and blood samples were collected at baseline and after three months	qPCR for rRNA-polymerase β subunit	↑ Lactic Acid Bacteria (*p*<0.05)	↓ Inflammatory cytokines ↓ Oxidative stress ↓ Myeloperoxidase ↓ 8-hydroxy-2-deoxyguanosine ↑ IL-10	NA	NA
							NA	NA			
							• Specific score from 0 to 18 for adherence				
**7**	**Pagliai et al. 2019 **[46]	A crossover study	23	Italy	over- weight individuals (16 F, 7 M) Mean age: 58.6 ± 9.8 y	healthy subjects were randomly assigned to isocaloric MD or VD diets lasting 3-months each and then crossed	16S rRNA sequencing	*↑Lachnoclostridium (p=0.039)* *↑Enterorhabdus (p=0.003)* *↑Parabacteroides (p=0.037)* *↑Clostridium sensu stricto (p=0.005)* *↑Veillonella (p=0.029)* *↓Anaerostipes (p=0.048)*	↓ Inflammatory cytokines: ↓VEGF, ↓MCP-1, ↓IL-17, ↓IP-10 ↓IL-12,	**Alpha** diversity via Simpson and Shannon/ no significant correlation **Beta** diversity via UniFrac and Bray-Curtis/ no significant distinction	↑fruit, ↑vegetables, ↑cereals, ↑legumes, ↑olive oil $\to$ fish $\to$ poultry $\to$ dairy products ↓ red meat ↓ wine
							GC-MS	↑ propionic acid (*p*=0.034); But no effect on butyrate, acetate, isobutyrate, isovalerate or valerate			
							NA				
**8**	**Ghosh et al. 2020 **[47]	Randomized, multicenter, single-blind,	612	UK, France, Netherlands, Italy, Poland	non-frail or pre-frail elder subjects	Gut microbiota before and after the administration of a 12 month long MedDiet intervention tailored to elderly subjects	16S rRNA sequencing	↑*Faecalibacterium* ↑*Roseburia* ↑*Eubacterium* ↑*Bacteroides* ↑*Prevotella* ↓*Anaerostipes* ↓*Ruminococcus* ↓*Collinsella* ↓*Coprococcus* ↓*Dorea* ↓*Clostridium* ↓*Veillonella* ↓*Flavonifractor* ↓*Actinomyces* ↑*F. prausnitzii,* ↑*R. hominis* ↑*E. rectale* ↑*E. eligens* ↑*E. xylanophilum* ↑*B. thetaiotaomicron,* ↑*P.copri* ↑*A.hadrus* ↓*R. torques* ↓*C. aerofaciens* ↓*C. comes* ↓*D. formicigenerans* ↓*C. ramosum* ↓*V. dispar* ↓*F. plautii* ↓*A. lingnae* (*p*-values were not mentioned)	Lower frailty; Improved cognitive function; ↓Inflammatory markers; ↓CRP ↓IL-17	**Alpha** diversity via undetermined index/ no significant correlation	↑Fruits ↑Vegetables ↑Wholegrains ↑Legumes ↑Fish ↓Fats ↓Alcohol ↓Sugar
							NA	↑ SCFA ↓ Secondary bile acids, ↓ p-cresols, ↓ ethanol ↓ carbon dioxide (*p*-values not mentioned)			
							NA				
**9**	**Pisanu et al. 2020 **[48]	Randomized controlled ntervention study	69	Italy	Case group: 23 obese/overweight patients with BMI > 25 and being “diet-free” as defined. (20 F, 3 M) Mean age: 53±9 years Control group: 46 individuals normal weight being “diet-free” as defined. (40 F, 6 M) Mean age: 49±11 years	The Gut Microbiota of Obese and overweight patients was compared before (T0) and after 3 months (T3) of nutritional intervention by MD	16S rRNA sequencing	*↑* Bacteroidetes ↑ Proteobacteria ↓ Firmicutes *↑ Sphingobacteriaceae* *↑ Sphingobacterium* *↑ Bacteroides* *↑ Prevotella stercorea* *↑ Proteobacteria* *↓ Lachnospiraceae* *↓ Ruminococcaceae* *↓ Ruminococcus* *↓ Veillonellaceae* *↓ Catenibacterium* *↓Megamonas* *↓Sutterella* (*p*-values<0.05*)*	Body weight ↓ Fat mass ↓	**Alpha** diversity via Shannon/ no significant correlation **Beta** diversity via Bray-Curtis/ no significant distinction between control and case group after intervention. **Only significant at baseline between case and control.**	*↑*Vegetables *↑*fruit *↑*cereals *↑*fish *↑*pulses
							NA	NA			
							• MDS (0 to 55)				
**10**	**Zhu et al. 2020 **[49]	Pilot study	10	USA	Healthy subjects 18-25 years old, Mean age: 22.1 y Mean BMI: 24.39	Fast food diet for 4 days followed by Mediterranean diet for 4 days, with a 4-day washout in between	16S rRNA sequencing	*↓ Collinsella* (*p*=0.028) *↑ Butyricicoccus* (*p*=0.019)	NA	**Alpha** diversity via undetermined assessor/ no significant correlation **Beta** diversity via UniFrac/ no significant distinction	NA
							LC-MS	↑Beneficial metabolites: ↑ indole-3-lactic acid (*p*=0.003) ↑indole-3-propionic acid (*p*<0.001)			
							NA				
**11**	**Galié et al. 2021 **[34]	Randomized controlled intervention	50	Spain	Metabolic Syndrome patients without T2DM/ any other PMH/DH Mean age: 51.37 y (25-60) Mean weight: 85.1 BMI: 25-35	adults with Metabolic Syndrome were randomized to a controlled, crossover 2-months dietary-intervention trial with a 1-month wash-out period, following a MedDiet or consuming nuts	16S rRNA sequencing	*Lachnospiraceae↑ (p<0.05)* *Ruminococcaceae↑ (p<0.05)*	Glucose↓ Insulin↓ HOMA-IR↓	**Alpha** diversity via Phyloseq/ no significant correlation **Beta** diversity via Bray-Curtis/ no significant distinction	↑Vegetables ↑fruit ↑cereals ↑fish ↑Olive oils ↓Red meat ↓Butter ↓ Sugary beverages
							LC-MS	↑ homocitrulline ↑ byacetate, ↑ cadaverine ↑ malate (*P* values<0.05)			
							17 items MDS				
**12**	**Galié et al. 2021 **[35]	Crossover randomized clinical trial	44	Spain	Metabolic Syndrome patients without T2DM/ any other PMH/DH Age range: 37-65. BMI: 25-35	crossover 2-months dietary-intervention trial with a 1-month wash-out period, consuming a MD or a non-MD plus nuts. Nutritional data were collected at the beginning and the end of each intervention period using 3-day dietary records	16S rRNA sequencing	No significant changes in the characteristics and composition of gut microbiota (*p*>0.05).	Glucose↓ Insulin↓ HOMA-IR↓	NA	↑Vegetables ↑fruit ↑cereals ↑fish ↑Olive oils ↓Red meat ↓Butter ↓ Sugary beverages
							GC-MS LC-MS	↑ HpEPE, ↑ testosterone, ↑ PC ↑ TMA, ↑ succinic acid, ↑ ChoE ↑ taurolithocholic acid, ↑ amino acids ↑ LPC ↑ carnitine species ↑ TG ↑ LPE (*p*-values<0.05)		NA	
							• 17-item MDS				
**13**	**Ismael et al. 2021 **[39]	Single-arm pilot study	9	Portugal	9 patients with type 2 diabetes (3F and 6M) Age range: 47-77 years Mean age: 66 ± 9 years Mean BMI of 27.60 ± 4.03 Kg/m^2^	12-week single-arm pilot study, participants received individual nutritional counseling sessions; indices were assessed at baseline, 4 weeks, and 12 weeks after the intervention	16S rRNA sequencing	*Prevotella* to *Bacteroides* ratio↑ (*p*=0.438) *Firmicutes* to *Bacteroidetes* ratio↑ (*p*=0.846) Total Gut bacteria↑	↑ Glycemic control ↓ HbA1c ↓ HOMA-IR	**Alpha** diversity via Chao1 and Shannon/ no significant correlation **Beta** diversity via Bray-Curtis/ no significant distinction	Vegetables fruits; grains cereals; fish Legumes
							NA	NA			
							• MEDAS score				
**14**	**Muralidharan 2021 **[50]	Randomized controlled trial	400	Spain	Overweight/obese subjects Age range: 55–75 years BMI: 27-40 200 in IG 200 in CG	IG: intensive weight loss lifestyle intervention based on an energy- restricted, MD and physical activity CG: non-energy- restricted MD For one year	16S rRNA sequencing	*Firmicutes* ↑*↓* (*p*=NA) *Lachnospiraceae*↑ (*p*-value<0.001) *Butyricicoccus ↓* *Haemophilus ↓* *Ruminiclostridium ↓* *Eubacterium hallii ↓* (*p*-values<0.05)	↓ BMI ↓ HbA1C ↓ FBS ↑ HDL	**Alpha** diversity via Chao1 and Shannon/ no significant correlation **Beta** diversity via Bray-Curtis and UniFrac/ no significant distinction	NA
							NA	NA			
							• 17-item MDS				
**15**	**Rejeski et al. 2021 **[51]	pilot study of controlled diets	10	USA	Healthy individual without PMH/DH; 4F, 6M Mean age:31.8 y Mean BMI:22.9	Subjects gave a stool sample at baseline and then was provided with prepared meals of a “typical” American diet; after 2 weeks, a second stool sample was collected. All subjects were then provided with prepared meals based on the MD for another 2 weeks, followed by a final stool sample collection.	16S rDNA sequencing	*↑ Akkermansia,* *↑ Lactococcus,* *↑ Lachnospira* Ratio of *Firmicutes/Bacteroidetes ↑* *↓ Coprococcus* (*p*-values<0.05)	NA	**Alpha** diversity via Simpson/ **significantly increased** **Beta** diversity via Bray-Curtis/ no further association.	Fruits Vegetables
							NA	NA			
							NA				
**16**	**Barber 2021 **[52]	cross-over, randomised study	18	Spain	Healthy individual without Gastrointestinal PMH; Age range: 18–38 BMI range: 19.2–25.5	Each diet (Western-type diet and fibre-enriched MD) was administered for 2 weeks preceded by a 2-week washout diet	DNA quantification	↑ *Anaerostipes hadrus* *↑ Agathobaculum butyriciproducens* (*p*-values<0.05)	↑ Gas Evacuation number and volume ↑ Bowel Movement	**Alpha** diversity via Simpson, Shannon, Chao1, inverse Simpson/ no significant correlation **Beta** diversity via Bray-Curtis/ **significant association**	↑fruits, ↑vegetables ↑legumes
							LC-MS	↑ deoxycholate Glucuronide ↑ 5-hydroxyindole ↑ L-aspartyl-L-phenylalanine ↑ TMAO (*p*-values<0.05)			
							NA				
**17**	**Choo et al. 2023 **[42]	cross-over, randomised study	34	Australia	age between 45 and 75 years; Adults with SBP $\ge$ 120mmHg and risk factors for cardiovascular disease; not on hypertensive medication	Patients were randomly assigned to a MD or low-fat control diet for 8 weeks. patients underwent an 8-week washout period	16S rRNA sequencing	*↑Butyricicoccus* *↑Lachnospiraceae* *↑Streptococcus* *↓Colinsella* *↓Veillonella* (*p*-values<0.05)	*↓* SBP ↑FBS	**Alpha** diversity via Faith/ no significant correlation	↑fruits, ↑vegetables ↑legumes ↑cereals
							NA	NA		**Beta** diversity via Weighted Unifrac/ no significant association	
							10-point and 18-point MDS				
**18**	**Boughanema et al. 2023 **[36]	Single arm trial	91	Spain	91 Patients with obesity and metabolic syndrome; BMI≥ 27 and ≤40 kg/ m2	Patients were stratified as Low or optimal vitamin D groups on baseline. Both received a hypocaloric MD regimen for one year.	16S rRNA sequencing	↑Bacteroidetes ↑Firmicutes ↑Proteobacteria (*p*=0.002 for all three phyla)	*↓* Wight *↓*BMI (in optimal vitamin D group) *↓*HbA1C (in optimal vitamin D group) ↑HDL (only in low vitamin D group)	**Alpha** diversity via Faith-PD and Chao1/ significant correlation	NA
							NA	↑butanoate (*p*=0.018)		**Beta** diversity via Weighted and Unweighted Unifrac/ significant distinction	
							NA				
**19**	**Gomez-Perez et al. 2023 **[40]	Single arm trial	297	Spain	NAFLD or NASH cases; Men aged 55-75 and women aged 60-75; BMI≥ 27 and ≤40 kg/ m2; patients with a history of CVD or chronic condition were excluded.	participants were stratified into three groups according to alterations in the Hepatic Steatosis Index (HSI) or the Fibrosis−4 score (FIB−4) between baseline and after one year of intervention by MD	16S rRNA sequencing	*↑Alcaligenaceae* *↑Bifidobacteriaceae* *↓Proteobacteria* *↓Lentisphaerae* *↓Enterobacteriaceae* *↑Bifidobacterium* *↑Faecalibacterium* *↑Sutterella* *↑Desulfovibro* *↑Lachnospira* *↑Oscillospira* *↓Blautia* (*p*-values<0.05)	*↓*HbA1C	**Alpha** diversity via Faith-PD and Shannon/ No significant correlation	NA
							NA	NA		**Beta** diversity via Weighted Unifrac/ significant distinction	
							NA				
**20**	**Shoer et al. 2023 **[53]	Single blinded randomized control trial	200	Israel	Age range: 18-65 Exclusion criteria: • Use of diabetes medications, • Use of antibiotics three months before enrollment • Chronic diseases, or chronic use of medications that affect glucose/energy metabolism or HbA1c	200 participants were randomly assigned to a ratio of 1:1 to MD and PPT regimens for 6 months then followed for another 6 months; Participants met two of four glycemic criteria.	DNA quantification	*↑Ruminococcaceae* *↑Clostridiaceae* *↓Eubacteriaceae* *↑F. prausnitzii* *↓Eubacterium ventriosum* (*p*-values<0.05)	↑ Glycemic control ↑ Lipid control	**Alpha** diversity via Shannon index/significant correlation after MD intervention	↑whole-wheat bread and grains ↑legumes ↑fruits ↑vegetables, ↑olive oil ↑fish ↑poultry ↑low-fat dairy products
							LC-MS	27 metabolites significantly increased (*p*<0.05) and no metabolites significantly decreased: • 10 uncharacterized biochemical • 7 lipids • 6 amino acids, • xenobiotic (3-bromo-5-chloro-2,6-dihydroxybenzoic acid) • peptide (HWESASXX), • nucleotide (dihydroorotate) • bilirubin		NA	
							NA				

*SH *Surgical History, *LFHCC *Low-fat, high-complex carbohydrates diet, *MetS *Metabolic Syndrome, *CHD *Coronary Heart Disease, *FF *Fast Food, *PMH *Past Medical History, *DH *Drug History, *T2DM *Type-2 Diabetic Mellitus, *MetS *Metabolic Syndrome, *PC *Phosphatidylcholines, *TMA *Trimethylamine, *ChoE *Cholesterol esters, *LPC *Lysophosphatidylcholines, *LPE *Lysophosphoethanolamine, *HpEPE *Hydroxyyperoxide-eicosapentanoic acid, *TG *Triglycerides, *SCFA *Short Chain Fatty Acids, *IG *Interventional Group, *CG *Control Group, *MD *Mediterranean Diet, *TMAO *Trimethylamine-N-oxide, *SBP *Systolic Blood Pressure, *PPT *Postprandial glucose-targeting diet, *GC-MS *Gas Chromatograohy- Mass Spectrometry, *LC-MS *Liquid Chromatography-Mass Spectrometry, *MEDAS *Mediterranean Diet Adherence Screener

### Study characteristics

#### Observational studies

**Fig. 2:**
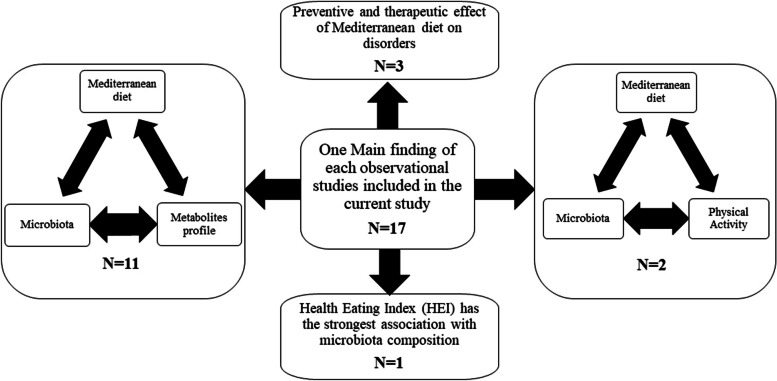
One main finding of each observational study included in the current study

The findings of competent observational studies are demonstrated in Table 1. Of 17 included observational studies, 6 were cohort studies, and 11 were cross-sectional. The total number of patients in 16 observational studies was 7838. In 14 studies, the effects of MD on healthy patients were evaluated [1, 11, 15, 17, 22–31]**.** In a study by Cox et al., the impact of MD on cirrhotic patients alongside healthy patients was assessed [14]. Moreover, one study was conducted on senior patients with a high prevalence of cardiovascular diseases [32]. At last, a recently published study by Calabrese et al. was conducted on patients with nonalcoholic fatty liver disease (NAFLD) [33]. Studies were conducted in Italy, Spain, Greece, the USA, Egypt, the UK, Turkey, and Canada. The USA was the most frequent country with five articles [14, 15, 23, 30, 32]. The mean age of patients in observational studies was 33.97 years. Of 7640 cases with available gender distribution, 39.62% were male. In 15 observational studies, 16srRNA/DNA sequencing was performed to determine gut microbiota composition [1, 11, 14, 15, 17, 22–26, 28–30, 32, 33]. Two studies did not specify the microbiota assessment method [27, 31]. The main findings of observational studies are summarized in Fig. 2.

#### Interventional studies

The findings of eligible interventional studies are summarized in Table 2. Of 20 interventional studies, three on metabolic syndrome [34–36], two were conducted on patients with Coronary Heart Disease [37, 38], one on type-2 diabetes mellitus [39], one on NAFLD or nonalcoholic steatohepatitis (NASH) cases [40], one on healthy patients with increased risk of developing colon cancer by definition [41], one on healthy individuals with risk factors for cardiovascular diseases [42], and other eleven studies were conducted on healthy patients [43–53]. Of eleven studies on healthy patients, five were conducted on patients with normal BMI [47, 49, 51–53], three on obese/overweight patients [44, 46, 50], and three other studies were conducted on both normal and elevated BMI patients in two different groups [43, 45, 48]. The total number of patients in interventional studies was 2402.

**Fig. 3:**
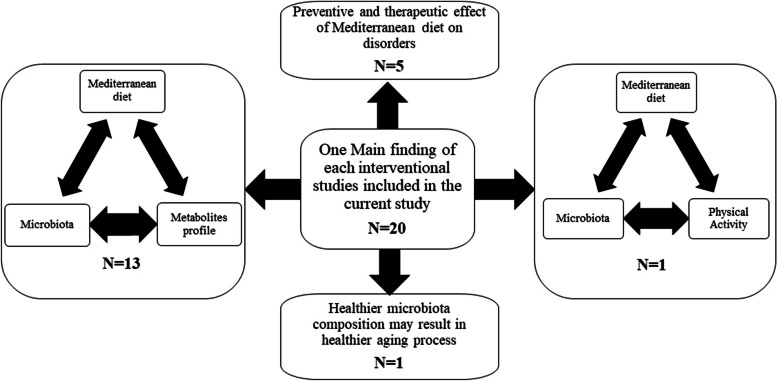
One main finding of each interventional study included in the current study

Of 20 interventional studies, nine were conducted in Spain [34–38, 40, 44, 50, 52], three in the USA [41, 49, 51], three in Italy [45, 46, 48], one in France [43], one in Australia [42], one in Portugal [39], one in Israel [53], and one was conducted on elders from five different centers in the world [47]. Of 20 interventional studies, one study was designed as triple arms [43], 11 studies as double arms [34, 35, 37, 38, 41, 44–46, 50, 52, 53], and seven studies as single arms [36, 39, 40, 47–49, 51]. Furthermore, five studies were designed as crossed-over trials [34, 35, 42, 46, 52]. Of 20 interventional articles, the 16S rRNA/DNA sequencing was carried out to identify gut microbiota composition. In the other four articles, either the method was not specifically mentioned or other methods were employed [43, 45, 52, 53]. The main findings of interventional studies are summarized in Fig. 3.

### Quality assessment and risk of bias

Of 17 observational studies' quality assessed by the adapted New-Castel Ottawa scale, five studies had a moderate quality score (4-6) [17, 25, 26, 28, 32]. Twelve other studies had a high-quality score ($\ge$ 7) [1, 11, 14, 15, 22–24, 27, 29–31, 33]. Data on observational studies' quality assessments are summarized in Tables 3 and 4.

**Table 3:** Adapted Newcastle-Ottawa assessment scale for cross-sectional studies

**No**	**Study**	**Selection**				**Compatibility**	**Outcome**		**Total**
		**Representativeness of cases**	**Sample size **^a^	**Non-respondents **^b^	**Ascertainment of the exposure**	**Potential confounders**	**Assessment of the outcome**	**Statistical test**	
maximum		5				1	3		9
		1	1	1	2	1	2	1	
1	Gutierrez-Diaz et al. 2016 [17]	1	0	1	2	1	0	1	6
2	Gutierrez-Diaz et al. 2017 [22]	1	1	1	2	1	0	1	7
3	Mitsou et al. 2017 [1]	1	1	1 (Drop:4)	2	1	0	1	7
6	Garcia-Mantrana et al. 2018 [25]	1	0	1	2	1	0	1	6
8	Cox et al. 2019 [14]	1	1	1	2	1	0	1	7
9	Gallè et al. 2020 [26]	1	1	0 (Drop: 104)	2	1	0	1	6
10	Maldonado-Contreras et al. 2020 [32]	1	0	1	2	1	0	1	6
11	Ruiz-Saavedra et al. 2020 [27]	1	1	1	2	1	0	1	7
12	Valeriani et al. 2020 [28]	1	1	0 (Drop: 64)	2	1	0	1	6
13	Rosés et al. 2021 [29]	1	1	1	2	1	0	1	7
14	Wang et al. 2021 [30]	1	1	1	2	1	0	1	7

^a^sample size more than 50 cases was considered ideal

^b^Non-response rate less than 5% was considered ideal

**Table 4:** Adapted Newcastle-Ottawa assessment scale for cohort studies

**No**	**Study**	**Selection**				**Compatibility**		**Outcome**			**Total**
		**Representativeness of the exposed cohort**	**Selection of the non-exposed cohort**	**Ascertainment of exposure**	**Demonstration that outcome of interest was not present at start of study**	**On age**	**On other risk factors**	**Assessment of the outcome**	**Duration of follow-up**	**Adequacy of follow-up of cohorts**^a^	
maximum		4				2		3			9
		1	1	1	1	1	1	1	1	1	
4	Shankar et al. 2017 [23]	1	0	1	1	1	1	0	1	1	7
5	Bowyer et al. 2018 [24]	1	N/A^b^	1	1	1	1	0	1	1	7
7	Maskarinec et al. 2019 [15]	1	1	1	1	1	1	0	1	1	8
15	Turpin et. al 2022 [31]	1	N/A^b^	1	1	1	1	0	1	1	7
16	De Filippis et al. 2015 [11]	1	1	1	1	0	1	0	1	1	7
17	Calabrese et al. 2023 [33]	1	1	1	1	1	1	0	1	1	8

^a^Lost to follow-up less than 5% was considered ideal

^b^As the study only consisted of one exposed group in its design, this item is not applicable

We used the term Not Applicable in different sections of the interventional studies quality assessment tool if 1) the patients were informed of their allocation to their groups (†) or 2) participants' randomized allocation was performed, but further blinding was not applicable due to the nature of dietary interventions (‡) or 3) only one group was assessed before and after dietary intervention (single arm) (). Data on interventional studies' quality assessment are summarized in Table 5.

**Table 5:** Cochrane bias assessment tool for interventional studies

No.	Study	Random sequence generation (selection bias)	Allocation concealment (selection bias)	Blinding of participants and personnel (performance bias)	Blinding of outcome assessments (detection bias)	Incomplete outcome data (attrition bias)	Selective reporting (reporting bias)	Other bias
1	Kong et al. 2014 [43]	Unclear	N/A^c^	N/A^c^	N/A^c^	High risk ^a^	Low risk	No
2	Haro et al. 2015 [44]	Low risk	Low risk	N/A^d^	N/A^d^	Low risk	Low risk	No
3	Haro et al. 2016 [37]	Low risk	Low risk	N/A^d^	N/A^d^	Low risk	Low risk	No
4	Haro et al. 2017 [38]	Low risk	Low risk	N/A^d^	N/A^d^	Low risk	Low risk	No
5	Djuric et al. 2017 [41]	Low risk	Low risk	N/A^d^	N/A^d^	Low risk	Low risk	No
6	Luisi et al. 2019 [45]	Unclear	N/A^c^	N/A^c^	N/A^c^	Low risk	Low risk	No
7	Pagliai et al. 2019 [46]	Low risk	Low risk	N/A^d^	N/A^d^	Low risk	Low risk	No
8	Ghosh et al. 2020 [47]	Unclear	N/A^e^	N/A^e^	N/A^e^	Low risk	Low risk	No
9	Pisanu et al. 2020 [48]	Unclear	N/A^e^	N/A^e^	N/A^e^	Low risk	Low risk	No
10	Zhu et al. 2020 [49]	Low risk	Low risk	N/A^d^	N/A^d^	Low risk	Low risk	No
11	Galié et al. 2021 [34]	Low risk	Low risk	N/A^d^	N/A^d^	Low risk	Low risk	No
12	Galié et al. 2021 [35]	Low risk	Low risk	N/A^d^	N/A^d^	Low risk	Low risk	No
13	Ismael et al. 2021 [39]	Unclear	N/A^e^	N/A^e^	N/A^e^	Low risk	Low risk	No
14	Muralidharan et al. 2021 [50]	Low risk	Low risk	N/A^d^	N/A^d^	Low risk	Low risk	No
15	Rejeski et al. 2021 [51]	Unclear	N/A^e^	N/A^e^	N/A^e^	Low risk	Low risk	No
16	Babrber et al. 2021 [52]	Low risk	Low risk	N/A^d^	N/A^d^	Low risk	Low risk	No
17	Choo et al. 2023 [42]	Unclear	Low risk	N/A^d^	N/A^d^	Low risk	Low risk	No
18	Boughanema et al. 2023 [36]	Unclear	N/A^e^	N/A^e^	N/A^e^	Low risk	Low risk	No
19	Gómez-Pérez et al. 2023 [40]	Unclear	Low risk	N/A^d^	N/A^d^	Low risk	Low risk	No
20	Shoer et al. 2023 [53]	High risk^b^	Low risk	N/A^d^	N/A^d^	Low risk	Low risk	No

^a^lost to follow-up in five of 50 cases

^b^Eligible participants were invited to the study

^c^Not Applicable due to informed patients allocation to different groups

^d^participants Randomized allocation was performed; but further blinding was no applicable due to different dietary intervention

^e^Not applicable; as there was only one group assessed before and after dietary intervention (single arm)

### Mediterranean Diet and gut microbiota diversity

#### Observational studies

Of 17 observational studies, 10 reported a correlation between MD and alpha or beta diversity [11, 14, 15, 23–26, 29, 30, 32]. Alpha diversity explains the structure of bacterial richness (number of taxonomic groups) or evenness (distribution of the abundance of groups) of both [54], while beta diversity summarizes the degree to which bacteria differ from one another [55]. In other words, the alpha index evaluates intra-sample diversity, whereas the beta index assesses inter-sample diversity.

Of 10 studies with reported diversity, eight analyzed both alpha and beta diversity [11, 15, 24–26, 29, 30, 32], one study reported only alpha diversity [14], and one study reported only beta diversity [23]. Four (of nine) studies reported beta diversity via the Bray-Curtis measure [23, 26, 29, 30], four reported beta diversity via the UniFrac measure [11, 15, 24, 32], and one study did not specify the beta measure [25]. Five (of nine) studies reported alpha diversity via the Shannon index [14, 15, 26, 29, 30]; one study reported alpha through four measures: Chao1, OTUs, Simpson, and Shannon [24]; one study reported Shannon and Chao1, simultaneously [25]. Another study reported alpha as both Shannon and Faith PD measures [32], and another did not specify the method for alpha diversity measurement [11].

Four (of nine) studies with a report on beta diversity had a significant bacterial separation following MD adherence [15, 23, 24, 26], three did not show a significant correlation [11, 29, 30], one had a significant correlation with some of the Mediterranean dietary components, but did not mention the correlation with total MD [32]. The last study did not mention the outcome of the beta diversity assessment [25].

Four (of nine) studies with a report on alpha diversity did not yield a significant correlation with MD adherence [11, 26, 29, 30], whereas three reported that MD adherence resulted in higher bacterial diversity [14, 15, 24]. In one study, the correlation between MD adherence and alpha diversity was significant via the Shannon index yet insignificant through Faith's PD [32]; another study revealed a significant correlation via Chao1, yet insignificant via Shannon [25]. Microbiota diversity in observational studies is summarized in Table 1.

#### Interventional studies

Of 20 interventional studies, 14 investigated alpha and beta diversity [34, 36, 38–42, 44, 46, 48–52], and two study only assessed alpha diversity [47, 53]. Of 16 studies with a report on alpha diversity, 13 claimed no significant association between alpha diversity and MD adherence [34, 38–42, 44, 46–50, 52]. Only in three studies, there were a significant association between MD and alpha diversity [36, 51, 53]. Furthermore, of 14 articles with a report on beta diversity, nine did not report any significant separation utilizing beta diversity neither between case and control group nor within a group before and after intervention [34, 38, 39, 42, 44, 46, 49–51], three study reported a significant bacterial separation after MD intervention [36, 40, 41]. Another study reported a significant difference at baseline between the case and control group before any intervention occurred [48]. Finally, in one study, MD adherence significantly affected beta diversity in intervened cases compared to the control group [52]. Microbiota diversity in interventional studies is summarized in Table 2.

### Mediterranean Diet and different bacterial abundance

#### Observational studies

Of 17 observational studies, 16 reported significant effects of MD on microbiota composition, and the abundance of at least one bacterium at the phylum, genus, or species level differed between groups. Just one study did not have a significant finding (*p*-value>0.05), but still, they claimed that there was a trend toward increasing Firmicutes and decreasing Bacteroidetes with MD adherence [28]. However, in that study, the specific impact of physical activities on microbiota, instead of MD adherence, is delineated and the impact is even statistically significant (*p*<0.05). Of 16 studies with a significant report on microbiota abundance, an increase in *Faecalibacterium* genus was reported in four articles [22, 27, 30, 31]. Furthermore, four articles reported an increase in either Bacteroidetes phylum or *Bacteroides* genus [1, 17, 26, 30]. Four articles mentioned an increase in either *Prevotellacea* family or *Prevotella* genus [11, 17, 23, 32]. The results of observational studies regarding gut bacterial abundance are summarized in Table 1.

#### Interventional studies

Of 20 interventional studies, 17 reported a significant change in bacterial abundance after MD intervention in at least one bacterium at the phylum, genus, or species level. Only three studies failed to find a significant change in bacterial abundance [35, 41, 43]. *Prevotella*, either in genus or species level, was increased in four studies [38, 39, 47, 48] yet decreased in one study [44]. Nevertheless, in one of the four studies with a report on increase in *Prevotella*, the amount of increase was not statistically significant [39]. In four studies*, Faecalibacterium* was increased at the genus level [37, 38, 40, 47]. Firmiticus phylum was increased in three studies [36, 39, 51], whereas it decreased in one [48]. In one study, both increasing and decreasing trends were observed in members belonging to Firmicutes Phylum [50]. Results of interventional studies regarding gut bacterial abundance are summarized in Table 2.

### Effect of Mediterranean diet adherence on microbial metabolites

#### Observational studies

Of 17 observational studies, 11 reported a significant change in microbial metabolites in MD adherent participants [1, 11, 14, 17, 22, 23, 25, 27, 29, 32, 33]. Five articles reported a significant increase in main SCFAs following MD adherence [11, 23, 25, 27, 29]. Acetate was significantly increased in four studies [1, 11, 23, 25], while in one study, it was significantly increased via the MDS assessment tool yet decreased via HEI [32]. Propionate was increased remarkably in five studies [11, 17, 23, 25, 32]. Microbial metabolites in observational studies are summarized in Table 1.

#### Interventional studies

Nine of 20 interventional studies showed significant changes in microbiota-derived metabolites following Mediterranean dietary intervention [34–36, 44, 46, 47, 49, 52, 53]. One article mentioned a remarkable increase in the concentration of SCFAs [47], and two reported a significant increase in propionic acid following MD [46, 49]. Microbial metabolites in interventional studies are summarized in Table 2.

### Effect of Mediterranean diet adherence on clinical outcomes

#### Observational studies

Four studies reported a significant clinical or clinical-related laboratory outcome [1, 14, 27, 33]. An increase in fecal moisture and defecation frequency, a decrease in bloating [1], decreasing a 90-day hospitalization risk [14], better glycemic/hyperlipidemic state control [33], and decreasing serum IL-8 level [27] were clinical outcomes mentioned in observational studies.

#### Interventional studies

In total, 15 studies reported a significant clinical outcome following dietary intervention [34–36, 39, 40, 42–48, 50, 52, 53]. Lowering inflammation was reported in four articles [43, 45–47]. Optimized diabetic control was reported eight times [34–36, 39, 40, 44, 50, 53]. Decreasing fat mass was reported four times [36, 48, 50, 53], lowering systolic blood pressure reported once [42], and better bowel movement was reported once [52].

## Discussion

This systematic review aimed to summarize the results of observational and interventional studies that examined the efficacy of the MD on the gut microbiota composition and clinical outcomes in different groups of people with distinct demographic characteristics and health statuses. This study reviewed 37 documents, divided into interventional and observational studies.

Consumption of the Mediterranean diet is associated with a different microbiota composition compared to Western-type dietary patterns. The microbiota composition associated with MD is characterized by higher microbial biodiversity. This characteristic of gut microbiota is defined as "α-diversity," demonstrating the number of species present in the microbiota and is associated with the health of individuals [54]. Besides, an intersample bacterial separation between two groups is measured through beta diversity [55]. In a study by Bowyer et al., Alpha diversity was significantly increased following MD adherence [24]. In another study by Maskarinec et al., alpha diversity was assessed in four dietary indices: HEI-2010, aHEI-2010, aMed, and DASH. Alpha diversity was increased significantly in tertiles in all four dietary indices [15].

Animal and human studies on gut microbiota composition via fecal samples have shown that all dietary changes could modulate gut microbial composition. In healthy subjects, a balanced diet can induce the formation of good microbial flora, which consists of all species of bacteria living in a system of control and mutual balance [56].

It is well established that gut microbial alteration may affect metabolism via secreted metabolites. The fermentation of the dietary components of the MD by the gut bacteria leads to the production of specific metabolites, such as SCFA, which is represented in the feces of subjects that follow MD [57]. SCFAs are carboxylic acids with six carbon atoms, maximumly, more frequently including acetic, propionic, and butyric acids [58].

Although the role of genetics in obesity is well known to everyone, human microbiota also plays a crucial role [59]. SCFA level, as the main metabolites of gut microbiota, is known to be altered in obesity as a result of dysbiosis, with more abundant Firmicutes relatively [60, 61]. Furthermore, SCFA alteration in obese patients results from increased *Lactobacillus* and *Staphylococcus* [62] and decreased *Bifidobacterium* [63].

The highest colon-rectal levels of SCFAs, specifically butyrate, would contribute to the reduced risk of CRC observed in Mediterranean countries. These protective effects could also contribute to the reduced presence of *Fusobacterium nucleatum*, which is mainly present in the colon of patients with CRC, and based on some related studies, it could be associated with the onset of this cancer [64]. Seven articles in our study reported increased SCFAs following MD (Tables 1 and 2).

On the other hand, TMAO (trimethylamine N-oxide) metabolites are present in higher concentrations in subjects that follow a Western diet [65]. Surprisingly, in a study by Barber et al., TMAO increased 1.5 times after MD. They speculated that ingesting choline-riched plant food, including legumes, prior to urinary sampling in the MD group might have been a reasonable explanation [52].

*Faecalibacterium prausnitzii*, a main known butyrate-producing bacteria with anti-inflammatory effects [66], was increased in seven studies [22, 27, 30, 37, 38, 47, 53], and no decrease in its level was reported in any of the included studies, despite the debate on impacts of MD on *F. Prausnitzii* level in the previous document [55]. Our findings were also parallel to those claiming MD may increase bacteria with polysaccharide affinity, including *E. Eligens* [30, 47], *Roseburia species* [29, 38, 44, 47], *Butyricicoccus species* [29, 49, 52] and also may decrease bacteria with simple sugars affinity [55], including *C. aerofaciens* [47]. Eventually, *Bacteroides* [1, 17, 26, 30, 37, 44, 47, 48] and *Parabacteroides* [37, 44] were among the most frequent microbiota species, which increased following MD adherence.

A significant effect on gut microbiota composition is believed to require long-term dietary pattern intervention. A study by Djuric et al. was conducted on the mucosal bacterial flora of the colon before or after six months of the Mediterranean or Western-type experimental diet. It revealed no significant differences in microbiota before or after the intervention [41]. Hence, a consistent dietary intervention should be considered for an almost permanent beneficial microbiota alteration.

In a study conducted on patients with metabolic syndrome, consuming the Mediterranean or traditional diet for two years, the MD has shown that it could partially reduce the typical dysbiosis of metabolic syndrome. The authors observed an increase in *Bifidobacterium* genera of the MD group [37].

Another outcome investigated in this review was the effect of the MD on the related clinical outcomes. In total, 19 studies investigated clinical outcomes after MD. The main reported outcomes were better diabetes mellitus management in nine articles, lowering inflammation in five articles, lowering fat mass in five, increasing bowel movement in one, and lowering hospitalization risk in one article (Tables 1 and 2).

Despite some controversies on the effect of SCFAs on inflammation [67], most studies in the literature delineated that SCFAs can decrease inflammation in the human body via inhibiting TNF-alpha and also upregulating IL-10 as an anti-inflammatory cytokine [68, 69]. Some authors also claimed that SCFAs can induce apoptosis, interrupt leukocyte migration, and inhibit the production of inflammatory mediators [70]. Besides, due to the epigenetic effects of SCFAs and their interaction with tissue receptors, their beneficial impacts on glucose homeostasis and decreasing glucose resistance have been proposed [71].

## Strengths and limitations

In this study, we thoroughly investigated observational and interventional studies, and clinical and microbiota alteration were assessed as outcomes simultaneously. However, most of the included studies in this systematic review had a limited number of participants, and only 15 of 37 articles investigated more than 100 participants in their research, which makes them heterogeneous in their design and representativeness. Various dietary assessment methods in observational studies and dietary interventions in interventional studies were utilized. Furthermore, some authors defined MD as a monounsaturated fatty acid-rich or enriched diet, while others adjusted this diet by adding nuts or other foods. Performing meta-analysis was impossible due to the included studies' heterogeneous nature.

## Conclusion

Adherence to the MD is associated with significant beneficial changes in the gut microbiota diversity, composition, and functions and major clinical improvements in most populations.

## Data Availability

No datasets were generated or analysed during the current study.

## Abbreviations

HEI: Healthy Eating Index
MDS: Mediterranean Diet Score
HFD-index: Healthy Food Diversity index
MD: Mediterranean Diet
NAFLD: Nonalcoholic fatty liver disease
NASH: Nonalcoholic steatohepatitis
SCFA: Short-Chain Fatty Acids
OTUs: Operational Taxonomic Units
AHEI-2010: Healthy Eating Index-2010
aMED: Alternate Mediterranean Diet
DASH: Dietary Approaches to Stop Hypertension Trial
Uni-Frac: Unique Fraction Metric
Faith's PD: Faith's Phylogenic Diversity
SCFAs: Short-chain fatty acids
TMAO: Trimethylamine N-oxide
CRC: Colorectal Cancer
PRISMA: Preferred reporting items for systematic reviews and meta-analyses
RA: Rheumatoid Arthritis
IBD: Inflammatory Bowel Disease
RCT: Randomized Clinical Trials
